# Generic Amplicon Deep Sequencing to Determine *Ilarvirus* Species Diversity in Australian *Prunus*

**DOI:** 10.3389/fmicb.2017.01219

**Published:** 2017-06-30

**Authors:** Wycliff M. Kinoti, Fiona E. Constable, Narelle Nancarrow, Kim M. Plummer, Brendan Rodoni

**Affiliations:** ^1^Biosciences Research Division, AgriBio, La Trobe UniversityMelbourne, VIC, Australia; ^2^AgriBio, School of Applied Systems Biology, La Trobe UniversityMelbourne, VIC, Australia; ^3^Department of Animal, Plant and Soil Sciences, AgriBio, La Trobe UniversityMelbourne, VIC, Australia

**Keywords:** *Ilarvirus* species, generic amplicon next-generation sequencing, virus genetic diversity, metagenomic next-generation sequencing

## Abstract

The distribution of *Ilarvirus* species populations amongst 61 Australian *Prunus* trees was determined by next generation sequencing (NGS) of amplicons generated using a genus-based generic RT-PCR targeting a conserved region of the *Ilarvirus* RNA2 component that encodes the RNA dependent RNA polymerase (RdRp) gene. Presence of *Ilarvirus* sequences in each positive sample was further validated by Sanger sequencing of cloned amplicons of regions of each of RNA1, RNA2 and/or RNA3 that were generated by species specific PCRs and by metagenomic NGS. *Prunus necrotic ringspot virus* (PNRSV) was the most frequently detected *Ilarvirus*, occurring in 48 of the 61 *Ilarvirus*-positive trees and *Prune dwarf virus* (PDV) and *Apple mosaic virus* (ApMV) were detected in three trees and one tree, respectively. *American plum line pattern virus* (APLPV) was detected in three trees and represents the first report of APLPV detection in Australia. Two novel and distinct groups of *Ilarvirus*-like RNA2 amplicon sequences were also identified in several trees by the generic amplicon NGS approach. The high read depth from the amplicon NGS of the generic PCR products allowed the detection of distinct RNA2 RdRp sequence variant populations of PNRSV, PDV, ApMV, APLPV and the two novel *Ilarvirus*-like sequences. Mixed infections of ilarviruses were also detected in seven *Prunus* trees. Sanger sequencing of specific RNA1, RNA2, and/or RNA3 genome segments of each virus and total nucleic acid metagenomics NGS confirmed the presence of PNRSV, PDV, ApMV and APLPV detected by RNA2 generic amplicon NGS. However, the two novel groups of *Ilarvirus*-like RNA2 amplicon sequences detected by the generic amplicon NGS could not be associated to the presence of sequence from RNA1 or RNA3 genome segments or full *Ilarvirus* genomes, and their origin is unclear. This work highlights the sensitivity of genus-specific amplicon NGS in detection of virus sequences and their distinct populations in multiple samples, and the need for a standardized approach to accurately determine what constitutes an active, viable virus infection after detection by molecular based methods.

## Introduction

The genus *Ilarvirus* is the largest in the *Bromoviridae* family, includes more than 20 recognized and tentative *Ilarvirus* species which are divided into six subgroups, and are characterized by a positive-sense, single-stranded tripartite RNA genome (Bujarski et al., 2012). Genomic RNA1 and RNA2 of all *Ilarvirus* species harbor genes that encode conserved proteins involved in viral replication. Genes on RNA3 encodes a movement protein (MP) and a coat protein (CP), which is expressed via sub-genomic RNA4 (Codoner and Elena, 2008; Pallas et al., 2012a). Many ilarviruses are transmitted by seed and pollen, and all are transmitted by vegetative propagation. Several *Ilarvirus* species also infect a wide plant host range within the family *Rosaceae*, including *Prunus* species, and can cause diseases of economic importance (Card et al., 2007; Pallas et al., 2012b).

Specific reverse transcription-polymerase chain reaction (RT-PCR) tests have been used widely for routine diagnosis of individual *Ilarvirus* species (Parakh et al., 1994; MacKenzie et al., 1997; Pallás et al., 1998; Scott et al., 2003). However, the infection of some plant hosts by several *Ilarvirus* species complicates species identification and necessitates the use of several different specific RT-PCR tests for detection. Furthermore, sequence diversity exists within *Ilarvirus* species (Kinoti et al., 2017), which can make the design of specific primers difficult and impact their detection by RT-PCR tests.

Broad spectrum degenerate primers based on conserved sequences within virus taxonomic groups such as family, genera and species, offer an alternative to the use of multiple species-specific RT-PCR tests for simultaneous detection of related viruses and unknown virus species (Compton, 1990; James et al., 2006; Maliogka et al., 2007). Sanger sequencing of the cloned generic PCR amplicons can then be used to identify the diversity of these virus groups. However, this approach to study virus diversity is usually limited to the sequencing of a few clones due to time, labor and cost constraints (Beerenwinkel and Zagordi, 2011). This limitation would hamper the detection of some low titers virus species and/or strains present in mixed infection when compared to higher titre virus species and/or strains.

NGS enable parallel sequencing of DNA from multiple samples at very high-throughput and at a high degree of sequence coverage generating large amounts of sequence data compared to Sanger sequencing of cloned PCR amplicons (Adams et al., 2009; Wu et al., 2015). However, most applications of NGS in plant virology are designed for virus discovery and full genome sequencing which frequently results only in consensus sequences of high occurring virus sequence variants and/or virus strains genomes (Adams et al., 2009; Radford et al., 2012; Prabha et al., 2013). In addition, most sample preparation methods lead to high background levels of host sequences compared to virus sequences associated during NGS (Marston et al., 2013; Hall et al., 2014). The consensus sequence and high levels of non-viral sequence associated with NGS offers limited resolution of low occurring sequence variant and/or strains within a virus isolate and also limits the number of samples that can sequenced in a single NGS run.

Next generation amplicon sequencing offers an alternative approach of an in-depth estimation of virus diversity and has been previously used to study the diversity of the *Ilarvirus Prunus necrotic ringspot virus* (PNRSV), within *Prunus* trees (Kinoti et al., 2017). An advantage of this approach is that it enables sequencing of virus amplicons from a high number of plant samples in a single run and can detect populations of both high and low-titre viruses within each sample (Wang et al., 2007; Eriksson et al., 2008). Until now, virus amplicon NGS has only been used to study population diversity within specific virus species (Eriksson et al., 2008; Beerenwinkel and Zagordi, 2011; Mancuso et al., 2011; Kinoti et al., 2017), unlike in bacteria where 16S ribosomal RNA gene amplicons are regularly deep sequenced to identify the diversity of bacterial species within an environmental sample (Sogin et al., 2006; Sanschagrin and Yergeau, 2014).

In this study, the novel approach of deep sequencing of genus-specific RT-PCR amplicons from a conserved region of the RNA2 encoded RNA-dependent RNA polymerase (RdRp) gene of *Ilarvirus* species was applied to 61 *Ilarvirus*-positive *Prunus* tree samples. Sequence analysis of resulting generic amplicon NGS data was used to determine the diversity of *Ilarvirus* species and strains infecting *Prunus* species in Australia.

## Materials and methods

### Sample extraction

Leaf tissue from 105 *Prunus* trees were collected in spring (2014–15) from five states of Australia (Table 1). Symptoms characteristic of virus infections were not noted at the time of sample collection. Total RNA was extracted from 0.3 g leaf tissue of each sample using the RNeasy® Plant Mini Kit (Qiagen) with a modified lysis buffer (MacKenzie et al., 1997).

**Table 1:** The number and Australian region of origin of *Prunus* samples used in this study.

**Region**	***Prunus* species**	**No. of samples**
New South Wales	Almond (*Prunus dulcis*)	35
Queensland	Apricot (*Prunus armeniaca*)	3
	Plum (*Prunus domestica*)	4
	Peach (*Prunus persica*)	6
	Nectarine (*Prunus nucipersica*)	1
	Sweet cherry (*Prunus avium*)	2
South Australia	Nectarine (*Prunus nucipersica*)	3
	Peach (*Prunus persica*)	1
Tasmania	Apricot (*Prunus armeniaca*)	1
	Almond (*Prunus dulcis*)	1
	Peach (*Prunus persica*)	5
	Plum (*Prunus domestica*)	2
	Sweet cherry (*Prunus avium*)	8
Victoria	Almond (*Prunus dulcis*)	27
	Peach (*Prunus persica*)	7
	Plum (*Prunus domestica*)	4
	Purple plum (*Prunus cerasifera*)	2

### PCR amplification

A 371 nucleotide (nt) region of the RNA2-encoded RNA-dependent RNA polymerase (RdRp) gene of ilarviruses was amplified from each sample using a previously published generic ramped annealing RT-PCR and nested PCR using *Ilarvirus* genus-specific degenerate primers (Maliogka et al., 2007). The RT-PCR was carried out using SuperScript® III One-Step RT-PCR System with Platinum® Taq High Fidelity (Invitrogen) for amplification of a 381 nt segment of the RdRp gene according to the manufacturer's instructions. The nested PCR reaction was performed using 1 μL of the first RT-PCR product with Platinum® Taq DNA Polymerase High Fidelity for amplification of a 371 nt segment of the RdRp gene according to the manufacturer's instructions. The PCR products were visualized by electrophoresis in 1.5% agarose gels stained with SYBR® Safe DNA gel stain (Invitrogen).

### Amplicon NGS library preparation

The *Ilarvirus* RNA2 RdRp amplicons were gel-purified using the Wizard® PCR clean-up kit (Promega) according to the manufacturer's instructions. Amplicon libraries were prepared and sequenced using the Illumina MiSeq as described previously (Kinoti et al., 2017). Briefly, in-house dual indexing adapter mpxPE2 consisting of unique barcodes for each amplicon were ligated to the 3′-terminus to incorporate the sequencing primer site, while an adaptor containing one of eight indexing sequences 5 bp in length (mpxPE1), was ligated to the 5′-terminus of each of the amplicons using the NEBNext® T4 ligase (New England BioLabs). PCR enrichment was carried out using an in-house multiplex PE barcode primer mix (2.5 μM) which were unique for each of the amplicons and Phusion® high fidelity PCR mastermix (New England BioLabs). PCR cycling conditions consisted of: one cycle at 98°C for 30 s, 15 cycles at 98°C for 10 s, 65°C for 30 s, 72°C for 30 s; and a final extension step at 72°C for 5 min.

After the PCR, excess primers and any primer dimer present were removed using Ampure XP® system (Beckman Coulter) according to the manufacturer's instructions. The size distribution and concentration of the amplicon libraries were determined using the 2200 TapeStation® system (Agilent technologies) and Qubit® Fluorometer 2.0 (Invitrogen), respectively, and the resulting quantification values were used to pool the amplicon libraries at equal concentration. The amplicon library was sequenced using the Illumina MiSeq with a paired read length of 301 base pairs. The amplicon sequence read data for this study have been submitted to the NCBI Sequence Read Archive (SRA) database under the Bioproject accession PRJNA380730 and SRA study accession SRP102631.

### Amplicon sequence reads analysis

The generated raw amplicon sequence reads were quality trimmed with a quality score of >20 and minimum read length of 200 nt using Trim Galore! (version 0.4.0) (Krueger, 2012) and paired using PEAR (version 0.9.4), with default parameters (Zhang et al., 2014). Cutadapt (version 1.4.1) (Martin, 2011) was used to trim each sample amplicon read to a similar size of 350 bp and the resulting amplicon sequence reads clustered/merged at 100% identity into distinct sequence variants clusters using Usearch (version 7.0.1090) (Edgar, 2010). To filter off any potential RT-PCR and sequencing error associated reads, sequence variants comprising of <10 individual reads were removed from the amplicon NGS dataset (Kinoti et al., 2017). Additionally, non-coding sequence variants were also removed because the generic-amplicons were each derived from *Ilarvirus* RdRp gene region.

In order to identify the *Ilarvirus* species that were detected by the *Ilarvirus* genus-specific PCR, the sequence variants were searched against published *Ilarvirus* RNA2 type isolate reference sequences in a local database (Table 2) using BLASTn with default parameters. Only sequence variants BLASTn matches with the highest identity and the highest bit score, were retained for sequence variants that had BLASTn matches to multiple *Ilarvirus* species.

**Table 2:** *Ilarvirus* RNA2 type isolate reference sequences to which the generic amplicon next generation sequencing data were mapped using BLASTn.

***Ilarvirus* species**	**GenBank accession No**.	**References**
*Ageratum latent virus isolate (AgLV)*	JX463341	Sharman and Thomas, 2013
*American plum line pattern virus (APLPV)*	AF235165	Scott and Zimmerman, 2000
*Apple mosaic virus (ApMV)*	AF174585	Shiel and Berger, 2000
*Asparagus virus 2 (AV-2)*	EU919667	Scott and Zimmerman, 2009
*Blackberry chlorotic ringspot virus (BCRV)*	DQ091194	Tzanetakis et al., 2010
*Blueberry shock virus (BLShV)*	KF031038	Rott et al., 2013, Unpublished
*Citrus leaf rugose virus (CiLRV)*	U17726	Ge and Scott, 1994
*Citrus variegation virus (CVV)*	EF584665	Li et al., 2008
*Elm mottle virus (EMoV)*	U57047	Ge et al., 1997
*Fragaria chiloensis latent virus (FClLV)*	AY707771	Tzanetakis and Martin, 2005
*Grapevine virus S (GVS)*	JX513899	Rott et al., 2012, Unpublished
*Lilac ring mottle virus (LiRMoV)*	FN669168	James et al., 2010
*Parietaria mottle virus (PMoV)*	AY496069	Scott et al., 2006
*Privet ringspot virus (PrRSV)*	KT290040	Aboughanem-Sabanadzovic et al., 2015
*Prune dwarf virus (PDV)*	AF277662	Rampitsch and Eastwell, 1997
*Prunus necrotic ringspot virus (PNRSV)*	AF278535	Di Terlizzi et al., 2001
*Raphanus latent virus (RaLV)*	JN107638	Perez-Egusquiza et al., 2014
*Spinach latent virus (SpLV)*	U93193	Scott et al., 2003
*Strawberry necrotic shock virus (SNSV)*	AY743591	Tzanetakis et al., 2010
*Tobacco streak virus (TSV)*	U75538	Scott et al., 1998

All *Ilarvirus* species variant sequences that were identified by the BLASTn analysis were pooled and aligned with all available RNA2 sequences (Table S3) for each *Ilarvirus* species available on GenBank, which were trimmed according to the corresponding RdRp RNA2 region amplified by the genus-specific degenerate primers using Muscle (version 3.8.31) (Edgar, 2004). A neighbor-joining phylogenetic tree was constructed using Phylip version 3.6 (Felsenstein, 2005). The resulting trees were visualized in FigTree version 1.4.2 (Andrew, 2009) and branches that had <90% bootstrap support were collapsed. Sequence pairwise identity comparison analysis was then carried out using the sequence demarcation tool (SDT) (version 1.2) (Muhire et al., 2014) on the aligned amplicon sequence variants of each sample.

### Sanger sequencing and metagenomics NGS of selected samples for validation of *Ilarvirus* species detected by the RNA2-generic amplicon NGS

To confirm the presence of the *Ilarvirus* species sequences detected by the generic amplicon NGS analysis, amplicons of ten samples selected were cloned using the pGEM®-T Easy vector system (Promega). Five clones of each of the 10 samples were sequenced using the SP6 and T7 promoter primers and ABI BigDye Terminator Version 3.1 kit on an AB3730xl sequencing machine (Applied Biosystems). The resulting sequences were subjected to a BLASTn search of NCBI database with default parameters (Altschul et al., 1997).

Specific PCR primers were designed to detect the same region of the RdRp gene amplified using the generic *Ilarvirus* nested PCR for each of the viruses that were identified by NGS (Table 3). Previously published and new PCR primer pairs were also used for RT-PCR amplification of either RNA1 and/or RNA3 segment of each *Ilarvirus* species/subgroup detected by the generic amplicon NGS (Table 3). RT-PCR was carried using the SuperScript™ III One-Step RT-PCR System (Invitrogen) as previously described with the appropriate temperature for each primer pair (Table 3). The RT-PCR amplicons were cloned, Sanger sequenced and the resulting sequences subjected to a BLASTn search as previously described.

**Table 3:** Specific and degenerate primers used for PCR amplification of either RNA1, RNA2 and/or RNA3 segment of each *Ilarvirus* species.

**Virus**	**Primers**	**Primer sequence (5′−3′)**	**Annealing temp**.	RNA (Gene^*^)	**Amplicon length**	**References**
*American plum line pattern virus* (APLPV)	APLPV- 2-F	AAATCACAGGGACGACTTCA	56°C	RNA2 (RdRp gene)	730 bp	This study
	APLPV- 2-R	TCCGACTCTTCCTACAATGC				
	APLPV3-F	GATCAGACGCTTTTGCAGTT	54°C	RNA3 (CP gene)	357 bp	This study
	APLPV3-R	CCTCCAGCTACCAAAACAGA				
*Apple mosaic virus* (ApMV)	ApMV-Rd-F	GCAGAGGTTGCATCATTTGA	55°C	RNA2 (RdRp gene)	347 bp	This study
	ApMV-Rd-R	GAAATTTGGCCTCAAAGTTG				
	ApMV-F	TGGATTGGGTTGGTGGAGGAT	53°C	RNA3 (CP gene)	261 bp	Petrzik and Svoboda, 1997
	ApMV-R	TAGAACATTCGTCGGTATTTG				
*Parietaria mottle virus* (PMoV)	PMoVf	GATGTTGCCGCCGACGATTCTA	54°C	RNA1 (MT gene)	475 bp	Janssen et al., 2005
	PMoVr	TTTTCCCACAACCCGCAACAC				
	PMoV-Rd-F	GATTGAAGAGTGATACGCAC	55°C	RNA2 (RdRp gene)	372 bp	This study
	PMoV-Rd-R	CAGGGTCGACTACACCACGAT				
*Prune dwarf virus* (PDV)	PDV-Rd-F	CGACGTTTGATAAGTCGC	54°C	RNA2 (RdRp gene)	360 bp	This study
	PDV-Rd-R	ATTTGGCTTCGAAATTGAAC				
	PDV-F	TAGTGCAGGTTAACCAAAAGGAT	62°C	RNA3 (CP gene)	172 bp	Parakh et al., 1994
	PDV-R	ATGGATGGGATGGATAAAATAAT				
*Prunus necrotic ringspot virus* (PNRSV)	PNRSV-Rd-F	GCAAAGGTTACATCACCTGATTC	60°C	RNA2 (RdRp gene)	363 bp	This study
	PNRSV-Rd-R	TTGGTTATGTGGAAATTTCGCTTC				
	PNRSV-C537	ACGCGCAAAAGTGTCGAAATCTAAA	54°C	RNA3 (CP gene)	455 bp	MacKenzie et al., 1997
	PNRSV-H83	TGGTCCCACTCAGAGCTCAACAAAG				
*Raphanus latent virus* (RaLV)	RaLV-Rd-F	AAGGGAAGTTACACCATGATG	52°C	RNA2 (RdRp gene)	347 bp	This study
	RaLV-Rd-R	GAATTTGGTTTCGAAATTAAAGA				
	RaLV-F	TGAGATCAGTTAACCGATGC	58°C	RNA3 (CP gene)	307 bp	This study
	RaLV-R	GCATCTATCAAGGTACCCAC				
*Ilarvirus* genus sub group 1	Ilar-grp1-MT-F	AWTCITCICAYAGTTTTGCYGC	52°C	RNA1 (MT gene)	870 bp	This study
	Ilar-grp1-MT-R	ATIGTIGCIATRTAITGVAC				
*Ilarvirus* genus sub group 1	Ilar-grp1-CP-F	TGATICCAAIGAIGCNAT	50°C	RNA3 (CP gene)	271 bp	This study
	Ilar-grp1-CP-R	TYIAGICACCAIACIAIDG				

* *MT = methyl transferase; RdRp = RNA dependent RNA polymerase; CP = coat protein*.

To sequence the full genome of the ilarviruses that were detected by generic amplicon NGS, 5 μl aliquots of total RNA extract from each of the 10 selected samples were used to prepare 10 metagenomic NGS libraries using NEBNext® Ultra™ RNA Library Prep Kit (New England BioLabs) following the manufacturer's instructions. The 10 libraries were sequenced using the Illumina MiSeq with a paired read length of 2 × 300 bp. De novo assembly of the resulting sequence reads into contigs using CLC Genomics Workbench and BLASTn analysis of the assembled contigs was carried out. The assembled virus contig sequences were submitted to GenBank and the accession number of each virus RNA sequences is included as supplementary information (Table S5).

## Results

### *Ilarvirus* RNA2 (RdRp) generic amplicon NGS data and BLASTn analyses

In total, amplicons of the expected size were obtained for 61 out of 105 samples tested with the *Ilarvirus* genus RNA2-specific nested PCR. A total of 10,381,728 total raw reads were generated following NGS of the 61 amplicon samples. After quality trimming, there were a total of 8,083,442 reads used for analysis, with an average of 132,515 reads per sample (Table S1). The quality trimmed amplicon reads were clustered at 100% identity, which resulted in an average of 33,819 sequence variants clusters per sample. Filtering/removal of singletons and sequence variant clusters comprising of <10 reads resulted in averages of 66,532 reads and 554 sequence variants per amplicon samples (Table S1).

BLASTn analyses of the clustered RNA2 amplicon sequence variants revealed the presence of four *Ilarvirus* species (PNRSV, PDV, APLPV, and ApMV) that have been previously reported in *Prunus* species and two potentially new *Ilarvirus* species amongst the 61 *Prunus* samples (Table 4; Table S2). PNRSV was the most frequently detected *Ilarvirus*, occurring in 48 of the 61 *Ilarvirus* RNA2-positive samples (Table 4; Table S2). PDV and APLPV were detected in three samples and ApMV was detected in one sample (Table 4; Table S2).

**Table 4:** Generic *Ilarvirus* species amplicon sequences detection summary.

***Ilarvirus* species**	**No. of positive samples**	**% range of amplicon sequence similarity to type isolate**	**% range of amplicon sequence coverage to type isolate**	**Type isolate GenBank Accession No**.
*American plum line pattern virus* (APLPV)	3	95–98	99–100	AF235165
*Apple mosaic virus* (ApMV)	1	97–99	98–100	AF174585
*Prune dwarf virus* (PDV)	3	92–99	97–99	AF277662
*Prunus necrotic ringspot virus* (PNRSV)	48	89–99	96–100	AF278535
*Ilarvirus*-S1	13	77–85	81–92	AY496069
*Ilarvirus*-S2	1	92–98	90–96	JN107638

RNA2 sequence variants of a putative *Ilarvirus* species that was detected in 13 peach tree samples of the 61 *Prunus* species samples had a most similar BLASTn match, with a sequence identity range of 77–82% and sequence query coverage of 81–87%, to the RNA2 of *Parietaria mottle virus* (PMoV) type isolate (GenBank accession AY496069) (Table 4; Table S2). Due to the low BLASTn identity and coverage to the RNA2 of the PMoV type isolate, the putative *Ilarvirus* sequences detected in these 13 samples have been tentatively named *Ilarvirus*-S1. Similarly, RNA2 sequences which had 92–98% identity and coverage of 90–96%, with *Raphanus latent virus* (RaLV) type isolate (GenBank accession JN107638), were detected in one cherry sample (Table 4; Table S2) and this putative *Ilarvirus* was tentatively named *Ilarvirus*-S2.

Mixed infections of PNRSV and APLPV was detected in three *Prunus* samples and one sample had a mixed infection of PNRSV and PDV. *Ilarvirus*-S2 which was only detected in one sample, also occurred in a mixed infection with *Ilarvirus*-S1. In samples with mixed *Ilarvirus* infections, one *Ilarvirus* species always occurred at a higher frequency, based on read numbers, compared to the other *Ilarvirus* species that were present in the sample (Table S2).

### Phylogenetic and sequence identity analysis

**Figure 1:**
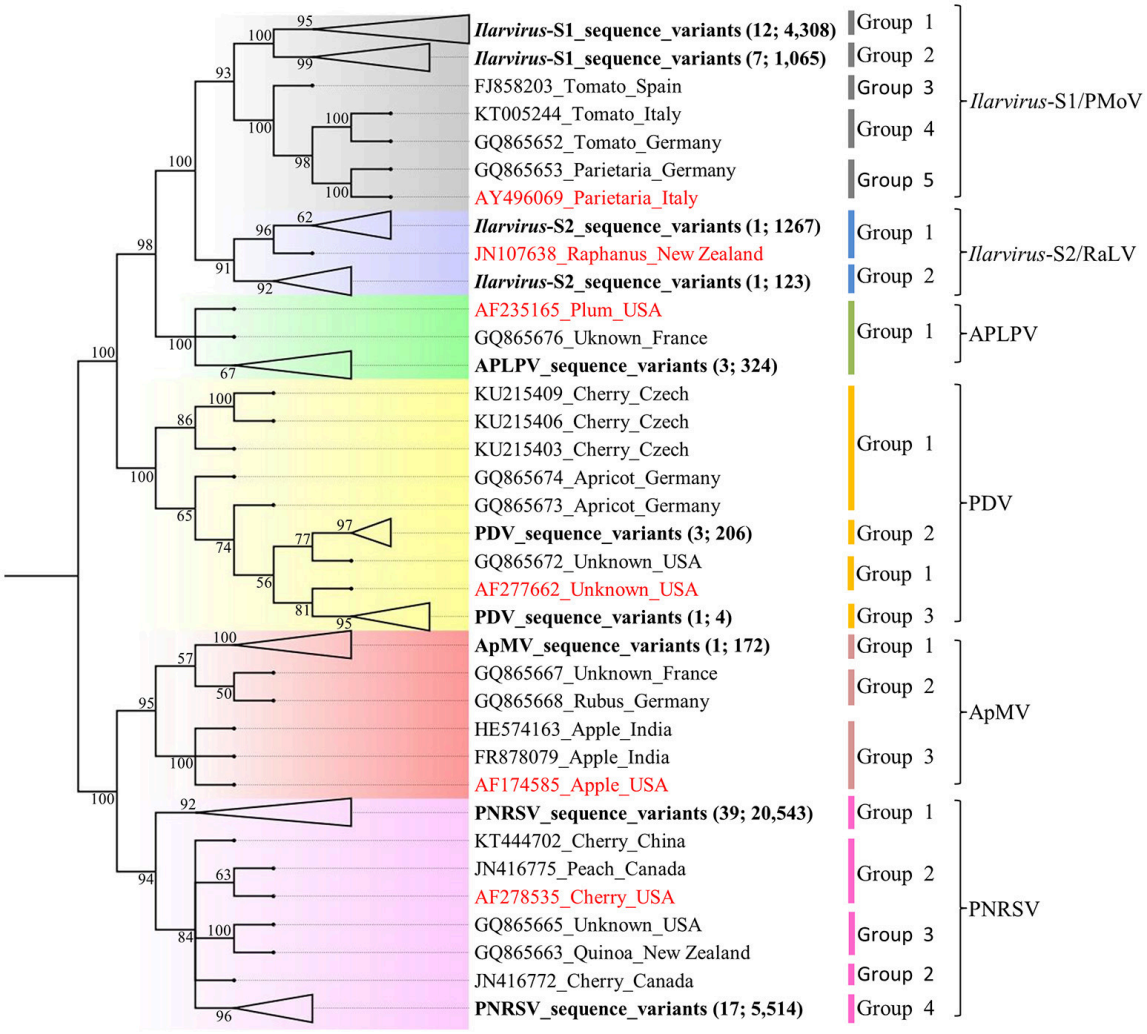
Neighbor-joining phylogenetic relationship of 324 *American plum line pattern virus* (APLPV), 172 *Apple mosaic virus* (ApMV), 210 *Prune dwarf virus* (PDV), 26,057 *Prunus necrotic ringspot virus* (PNRSV), 5,634 *Ilarvirus*-S1 and 1,390 *Ilarvirus*-S2 pooled sequence variants and the corresponding GenBank sequences of isolates of each virus (Table S3). The phylogenetic tree was constructed using Phylip version 3.6 with 1,000 bootstrap replicates and branches with <50% bootstrap support were collapsed. The branch position of the sequence variants of each *Ilarvirus* species from this study are in bold letters, the number of samples and variants are indicated in brackets and their branches collapsed for ease of presentation (Table 5). Each of the *Ilarvirus* type species is indicated in red font.

Phylogenetic analysis of the *Ilarvirus* species sequences detected in the 61 samples and GenBank isolate sequences, revealed that the Australian ApMV and APLPV sequence variants each formed one phylogenetic group and PNRSV, PDV, *Ilarvirus*-S1 and *Ilarvirus*-S2 sequence variants each formed two distinct phylogenetic groups. All phylogenetic groups had >90% bootstrap support which was used, along with a ≥96% identity criterion, as the minimum support required to define a distinct *Ilarvirus* phylogenetic group (Figure 1; Table 5). Phylogroups from *Ilarvirus*-S2 and APLPV phylogroup 1 were the only groups that included sequences from both GenBank and this study (Figure 1).

**Table 5:** The RNA2 phylogroups identified from phylogenetic analysis of pooled generic amplicon variant sequences from *Ilarvirus* species that were detected in 61 *Prunus* samples and the minimum percentage (%) sequence identity of sequence variants within each phylogroup.

***Ilarvirus* species**	**No. of positive *Prunus* samples**	**Australian *Ilarvirus* isolates sequence variants phylogroup**	**No. of *Prunus* samples positive for each phylogroup**	**No. of sequence variants**	**Minimum % sequence identity of sequence variants in each phylogroup**
*American plum line pattern virus* (APLPV)	3	1	3	324	96
*Apple mosaic virus* (ApMV)	1	1	1	172	97
*Prune dwarf virus* (PDV)	3	2	3	206	97
		3	1	4	99
*Prunus necrotic ringspot virus* (PNRSV)	48	1	39	20,543	96
		4	17	5,514	97
*Ilarvirus*-S1	13	1	12	4,308	96
		2	7	1,065	96
*Ilarvirus*-S2	1	1	1	1,267	97
		2	1	123	97

PNRSV, PDV, *Ilarvirus*-S1 and *Ilarvirus*-S2 each had one major phylogroup that was represented by more sequence variants and occurred in more of the plant samples compared to the other minor phylogroups for each virus (Figure 1; Table 5; Table S4). Seven of the 39 PNRSV-positive plant samples, one of the three PDV positive plant samples and seven of the 13 *Ilarvirus*-S1-positive plant samples had sequence variants occurring in two phylogenetic groups (Table S4). *Ilarvirus*-S2 was detected in a single cherry sample and its sequence variants diverged into two phylogroups (Figure 1; Table S4).

SDT identity analysis of amplicon sequence variants showed similar groupings as observed in the phylogenetic analysis (Figure S1). The sequence variants within each *Ilarvirus* species phylogroup had an identity of ≥96% (Table 5). However, in most samples, *Ilarvirus* sequence variants within a phylogroup had a higher percentage identity than the specified identity cut-off (Table S4). The distribution of pairwise identity between *Ilarvirus* sequence variants ranged between 90 and 99% for PNRSV, 94–99% for PDV, 97–99% for ApMV, and 96–99% for APLPV. *Ilarvirus*-S1 sequence variants had the widest distribution of pairwise identity with a range of 89–99% whereas the identity of *Ilarvirus*-S2 sequence variants ranged between 92 and 99% (Figure S1).

### Sanger sequencing and metagenomics NGS of selected samples for validation of *Ilarvirus* species detected by the RNA2-generic amplicon NGS

Sanger sequencing of the cloned *Ilarvirus* RNA2 generic amplicons confirmed the presence of some of the *Ilarvirus* species detected by the generic amplicon NGS in each of the ten samples. However, the PNRSV and PDV sequences in sample Pch4 and Ilarvirus-S1 and PNRSV sequences in sample Q15 that were identified by the *Ilarvirus* generic amplicon NGS, were not detected by cloning and sequencing of the *Ilarvirus* generic amplicon (Table 6).

**Table 6:** The viruses that were detected in each sample by amplicon NGS, cloning and sanger sequencing of *Ilarvirus* generic or virus specific PCRs and by metagenomics NGS in the ten *Prunus* samples that were analyzed further for the presence of ilarviruses.

**Sample**	**Prunus *specie***	***Ilarvirus* generic RNA2 amplicon NGS**	***Ilarvirus* generic RNA2 amplicon cloning & Sanger sequencing**	***Ilarvirus* RNA2 amplicon region specific RT-PCR & Sanger sequencing**	**RNA1/3 RT-PCR test & Sanger sequencing**	**RNA1/3 *Ilarvirus* subgroup 1 generic RT-PCR test**	**Metagenomic NGS**
BPch	*Prunus persica*	*Ilarvirus*-S1	*Ilarvirus*-S1 (371 bp)	*Ilarvirus*-S1 (372 bp)	–	–	–
Ch1	*Prunus avium*	*Ilarvirus*-S1	*Ilarvirus*-S1 (371 bp)	*Ilarvirus*-S1 (372 bp)	–	–	–
		*Ilarvirus*-S2	*Ilarvirus*-S2(371 bp)	*Ilarvirus*-S2(347 bp)	–	–	–
FPch	*Prunus persica*	*Ilarvirus*-S1	*Ilarvirus*-S1 (371 bp)	*Ilarvirus*-S1 (372 bp)	–	–	–
Pch2	*Prunus persica*	*Ilarvirus*-S1	*Ilarvirus*-S1 (371 bp)	*Ilarvirus*-S1 (372 bp)	–	–	–
Pch4	*Prunus persica*	*Ilarvirus*-S1	*Ilarvirus*-S1 (371 bp)	*Ilarvirus*-S1 (372 bp)	–	–	–
		PNRSV	–	PNRSV (363 bp)	PNRSV (455 bp)	NA	PNRSV full genome
		PDV	–	PDV (360 bp)	PDV (172 bp)	NA	PDV full genome
K75	*Prunus dulcis*	ApMV	ApMV (371 bp)	ApMV (347 bp)	ApMV (261 bp)	NA	ApMV full genome
M32	*Prunus dulcis*	PNRSV	PNRSV (371 bp)	PNRSV (363 bp)	PNRSV (455 bp)	NA	PNRSV full genome
NS9	*Prunus persica*	PDV	PDV (371 bp)	PDV (360 bp)	PDV (172 bp)	NA	PDV full genome
Q15	*Prunus armeniaca*	*Ilarvirus*-S1	–	*Ilarvirus*-S1 (372 bp)	–	–	–
		PNRSV	–	PNRSV (363 bp)	PNRSV (455 bp)	NA	PNRSV full genome
		APLPV	APLPV (371 bp)	APLPV (730 bp)	APLPV (357 bp)	NA	APLPV full genome
TAS3	*Prunus domestica*	*Ilarvirus*-S1	*Ilarvirus*-S1 (371 bp)	*Ilarvirus*-S1 (372 bp)	–	–	–

*(–) No amplicon result. NA, Not applicable; APLPV, American plum line pattern virus; ApMV, Apple mosaic virus; PDV, Prune dwarf virus; PNRSV, Prunus necrotic ringspot virus, and two putative Ilarvirus species*.

PNRSV, PDV, ApMV, APLPV, *Ilarvirus*-S1 and *Ilarvirus*-S2, were also detected using the specific RT-PCR tests. These tests were designed to detect the same RNA2 RdRp region as the generic *Ilarvirus* test in each of the samples in which they were detected by generic RNA2 amplicon NGS, and direct Sanger sequencing confirmed their identity (Table 6). Specific RT-PCR tests of the RNA1 and/or the RNA3 segment also detected PNRSV, PDV, ApMV and APLPV in each sample, in which these viruses had been detected by generic RNA2 amplicon NGS, and direct Sanger sequencing confirmed their identity (Table 6). However, *Ilarvirus*-S1 and *Ilarvirus*-S2 were not detected by the specific RT-PCR for PMoV RNA1 and RaLV RNA3, respectively (Table 6). Additionally, degenerate primers that were designed in this study to detect RNA1 and RNA 3 of subgroup 1 *Ilarvirus* species did not detect *Ilarvirus* sequences in any of the samples in which *Ilarvirus*-S1 and *Ilarvirus*-S2 RNA2 sequences were detected by generic amplicon NGS (Table 6).

The total raw reads obtained from the 10 plant samples by metagenomic NGS ranged from 1,192,869–5,489,161 and these numbers were reduced to 1,183,552–5,417,537 reads after quality trimming (Table S5). *De novo* assembly of reads from each individual plant sample resulted in 2,512–12,287 contigs across the 10 samples (Table S5). A BLASTn search of the GenBank database (Altschul et al., 1997) revealed contigs covering full genomes of PNRSV, PDV, ApMV and APLPV in samples in which they were detected by generic amplicon NGS. *Ilarvirus*-S1 and *Ilarvirus*-S2 RNA1, RNA2 and/or RNA3 sequences were not detected by metagenomic NGS in any of the ten plant samples despite the detection of their sequences by *Ilarvirus* RNA2 generic amplicon NGS (Table 6; Table S5).

## Discussion

NGS of *Ilarvirus* genus-specific PCR amplicons was used in this study to identify the diversity of *Ilarvirus* species detected in 61 *Prunus* trees in Australia. PNRSV, PDV, and ApMV which are known to infect Australian *Prunus* tree species, and APLPV, which has not been previously reported in Australia, were detected by this approach. Two novel and distinct groups of *Ilarvirus*-like RNA2 amplicon sequences were also identified in several trees by the generic amplicon NGS approach and these *Ilarvirus*-like sequences were tentatively named *Ilarvirus*-S1 and *Ilarvirus*-S2. The generic amplicon NGS approach used in this study was also sensitive enough to identify mixed infections of PNRSV and APLPV or PNRSV and PDV in four *Prunus* samples.

PNRSV was the most frequently detected *Ilarvirus*, occurring in 48 of the 61 *Ilarvirus* positive samples, but no particular *Prunus* host specificity was observed as described in other studies (Aparicio et al., 1999; Cui et al., 2015). In contrast, PDV was only detected in three peach tree samples and ApMV was only detected in one almond tree sample. Phylogenetic analysis identified distinct populations of sequence variants that formed two or more phylogroups within sequences representing PNRSV, PDV and ApMV (Figure 1; Table 5). A high bootstrap value of ≥90% was adopted as the lowest threshold required to confidently define a distinct RNA2 phylogroup for each *Ilarvirus* sequence variant population and these phylogroups were further confirmed by pairwise sequence identity comparison analysis (Kinoti et al., 2017). Phylogroups of PNRSV, PDV and ApMV sequence variants from this study did not include isolates from other countries that have been published in GenBank, which could indicate that Australian PNRSV, PDV, and ApMV isolates are distinct populations that may have evolved separately from isolates from other geographical regions. However, the limited number of *Ilarvirus* sequences available in GenBank for comparison with the Australian PNRSV, PDV, and ApMV sequence variants from this study, makes it difficult to explicitly conclude the observed phylogroup separation is associated with geographical origin.

APLPV was detected in three samples, representing the first report of APLPV detection in Australia. APLPV has been previously reported in North America, Europe and the Mediterranean region (Paulsen and Fulton, 1968; Myrta et al., 2002; Alayasa et al., 2003) but very little sequence information is available for comparison and analysis. Australian APLPV sequence variants and sequences of isolates from France and the USA formed a single phylogroup which indicated a low genetic variability of APLPV RNA2 RdRp sequences. Similar low genetic variability of the APLPV coat protein and movement protein gene regions on RNA3 was observed in other studies and did not differentiate between American and Mediterranean isolates nor plant host origins (Herranz et al., 2008).

The lack of APLPV sequence data and the lack of diversity observed in this study and in a study by Herranz et al. (2008), might reflect the low incidence and limited distribution of APLPV worldwide. However, symptoms associated with APLPV are similar to ApMV in *Prunus* hosts and it maybe that the virus is more widely distributed but remains undiagnosed due to the assumption that disease is caused by other viruses (Desvignes et al., 1999). The occurrence of distinct phylogroups in ApMV, PNRSV, PDV, *Ilarvirus*-S1 and *Ilarvirus*-S2 was further supported by pairwise identity analysis, with all *Ilarvirus* species phylogroups detected in this study having a general identity cut-off of ≥96%. Therefore, it is possible that these phylogroups represent putative genetic strains based on RNA2 and made up of a population of variants with ≥96% identity within the RNA2 component of ilarviruses detected in this study. Based on this assumption, this study identified two strains of PNRSV and PDV and only one strain for each of ApMV and APLPV occurring in Australia. The partial RNA2 RdRp segment used for analysis in this study is the most conserved region across all ilarviruses and it is possible more strains of these ilarviruses would be identified if similar analysis was carried out on the whole sequence of RNA2 component or the other genomic RNAs (Kinoti et al., 2017).

Other molecular based methods used in this study did not always confirm the presence of *Ilarvirus* species detected by the generic amplicon NGS. Sanger sequencing of the cloned *Ilarvirus*-generic RT-PCR amplicons only detected *Ilarvirus* species that occurred as a single infection or the *Ilarvirus* species with the highest amplicon NGS read numbers in mixed infections. For example, PNRSV and PDV occurred at a lower frequency (22.9 and 1.8% of amplicon NGS total read numbers, respectively) when compared to *Ilarvirus*-S1 sequences in sample Pch4 and these low frequency amplicon sequences were not detected when cloned generic amplicons were sequenced (Table 6). However, full genomes of PDV and PNRSV were assembled during metagenomic NGS, confirming their presence in Pch4. Therefore, it is possible that sequencing of cloned generic PCR amplicons may be limited in its detection of some virus species or strains with low frequency genomes in mixed infections compared to amplicon NGS approaches. This observed variation in species-specific numbers of the generic amplicons may be a result of varied PCR efficiency associated with the degenerate primer binding specificity to different virus species or variants which have previously been shown to negatively impact PCR amplicon copy numbers (Whiley and Sloots, 2005; Stadhouders et al., 2010).

The degeneracy associated with generic PCR primers make them more prone to non-specific amplification and formation of PCR artifacts due to mis-priming and diversity of nucleic acid extracts used for PCR (Qiu et al., 2001; Huber et al., 2009). For this reason, all the *Ilarvirus* species in each sample that were detected by NGS were tested by specific RT-PCR tests designed to amplify the same RdRp region as the generic *Ilarvirus* PCR (Maliogka et al., 2007). The detection of all expected *Ilarvirus* species confirmed that the generic amplicon NGS sequences were not from non-specific PCR products or artifacts. Virus species-specific RT-PCR also detected the other RNA components (RNA1 and 3) of all the *Ilarvirus* species expected in each sample with the exception of *Ilarvirus*-S1 and *Ilarvirus*-S2. *Ilarvirus*-S1 and *Ilarvirus*-S2 that were detected by NGS of the generic *Ilarvirus* RNA2 amplicons may represent two putative and novel *Prunus* infecting *Ilarvirus* species or strains. *Ilarvirus*-S1 and *Ilarvirus*-S2 sequences had greatest BLAST sequence identity to PMoV and RaLV, respectively, which belong to *Ilarvirus* subgroup 1 (Pallas et al., 2012b). However, the presence of a novel PMoV and RaLV related *Ilarvirus* could not be confirmed by the amplification of another region of the *Ilarvirus* RNA2 component nor by the amplification of RNA1 or RNA3. Nevertheless, RNA2 sequences of both putative ilarviruses, *Ilarvirus*-S1 and *Ilarvirus*-S2, underwent similar phylogenetic analysis as the known ilarviruses. *Ilarvirus*-S1 sequence variants formed two phylogroups that diverged from PMoV type isolate and *Ilarvirus*-S2 sequence variants formed two phylogroups.

Metagenomic NGS is widely used for plant virus detection and full genome characterization due to its sensitivity and lack of specificity compared to other molecular techniques (Petrosino et al., 2009; Mokili et al., 2012). Therefore, metagenomic NGS was used to confirm the presence of the *Ilarvirus* species detected by generic amplicon NGS in this study. Full genomes were assembled for PNRSV, PDV, ApMV, and APLPV. However, full genomes of *Ilarvirus*-S1 and *Ilarvirus*-S2, which have not been previously reported infecting *Prunus* species, were not assembled by the metagenomic NGS. The reason for the absence of *Ilarvirus*-S1 and *Ilarvirus*-S2 sequences when analyzed using the metagenomic NGS approach is not known, despite *Illavirus*-S1 and *Illarvirus*-S2 sequences being detected in large number based on generic amplicon NGS read numbers (Table S2). It is possible that *Ilarvirus*-S1 and *Ilarvirus*-S2 occurred in low titre in the plant, and the multiple PCR amplification steps during the generic nested PCR (Maliogka et al., 2007) coupled with preferential degenerate primer binding to *Ilarvirus*-S1 and *Ilarvirus*-S2 sequence may have resulted in their high generic amplicon NGS read numbers. It is also possible that the high background levels of host sequences compared to virus sequences associated with NGS (Hall et al., 2014), could have a negative impact on the detection of low titre virus infection by metagenomic NGS.

There is also a possibility that *Ilarvirus*-S1 and *Ilarvirus*-S2 virus sequences detected by generic amplicon NGS in this study may be derived from endogenous viral elements (EVEs). EVEs are composed of fragments or complete genome sequences of plant RNA viruses that are integrated in some plant and insect host genomes (Ndowora et al., 1999; Chiba et al., 2011; Cui and Holmes, 2012). These integrated viral sequences can have high sequence divergence from their related extant virus species that may have continued to evolve rapidly whilst the EVE has only evolved at the same rate as the host (Aiewsakun and Katzourakis, 2015). The *Ilarvirus*-S1 sequences detected in this study occurred mainly in peach trees and had a higher sequence divergence to PMoV type isolate, but their presence could not be confirmed by metagenomic NGS and therefore these *Ilarvirus*-S1 sequences could represent EVEs.

*Ilarvirus*-S2 sequences which were detected in only one sample were not highly divergent from RaLV (92–98% similarity) could also represent an EVE, especially as a full genome of this virus was not obtained via metagenomic NGS and no other sequences were amplified with RaLV specific PCR tests. Therefore, in this study it is difficult to conclusively determine whether the detection of *Ilarvirus*-S1 and *Ilarvirus*-S2 by generic NGS represent two potential new ilarviruses infecting *Prunus* species in Australia or are in fact EVEs. Further work is required to elucidate the origin of *Ilarvirus*-S1 and *Ilarvirus*-S2 amplicon sequences.

The lack of detection of *Ilarvirus*-S1 and *Ilarvirus*-S2 by metagenomic NGS in this study and also recently reported lack of NGS detection of some human viruses which were known to be present in reference material (Mee et al., 2016), raise serious concerns in the use of metagenomic and amplicon NGS as a “universal” virus diagnostic tool without understanding its detection limits and appropriate interpretation of NGS data. Similar concerns have also been raised by other authors regarding metagenomic NGS virus diagnostic sensitivity and what constitutes a virus infection (Martin et al., 2016). Currently, there is no standardized approach to accurately determine what constitutes an extant and active virus infection when analyzing an NGS dataset, especially where varied results and partial virus detection by molecular based methods is concerned, as in the case of *Ilarvirus*-S1 and *Ilarvirus*-S2 detection in this study.

This study highlights the potential of nested generic PCR to give false positive results for a virus, based on the detection of only a fraction of the genome, especially when an unknown virus or virus-related sequence is concerned. It is paramount that partial detection of virus sequences by molecular based methods be correlated to the presence of sequence from other RNA segments or region of the virus genome. Nevertheless, the findings of this study established that generic amplicon NGS approach is a highly suitable method for the rapid detection and identification of specific virus species from multiple plant samples and multiple virus infections in a single plant sample, all within one NGS run. Amplicon NGS allowed for the detection of known endemic and exotic *Ilarvirus* species in Australian *Prunus* species. The generic amplicon NGS analysis approach used in this study was also able to identify distinct RNA2 sequence variant populations, within and between plant samples, which could be identified as possible genetic strains of an *Ilarvirus* species.

## Author contributions

WK participated in the design of the study, collected and screened the samples for virus infection, carried out the library preparation and sequencing, performed computational analysis, and drafted the manuscript. FC participated in the design of the study, sample collection, data analysis, and contributed to drafting the manuscript. NN participated in collection and screening of samples for virus infection. BR participated in the design of the study, data analysis, and drafting the manuscript. KP participated in the design of the study, data analysis, and contributed to drafting the manuscript. All authors read and approved the final manuscript.

### Conflict of interest statement

The authors declare that the research was conducted in the absence of any commercial or financial relationships that could be construed as a potential conflict of interest.
