# Supplementary material for: Generic Amplicon Deep Sequencing to Determine Ilarvirus Species Diversity in Australian Prunus
Source: Front Microbiol. 2017 Jun 30;8:1219. doi: 10.3389/fmicb.2017.01219 (PMC5491605; doi:10.3389/fmicb.2017.01219)
Supplement: Supplementary file 1 [file Presentation1.PPTX]

## Slide 1
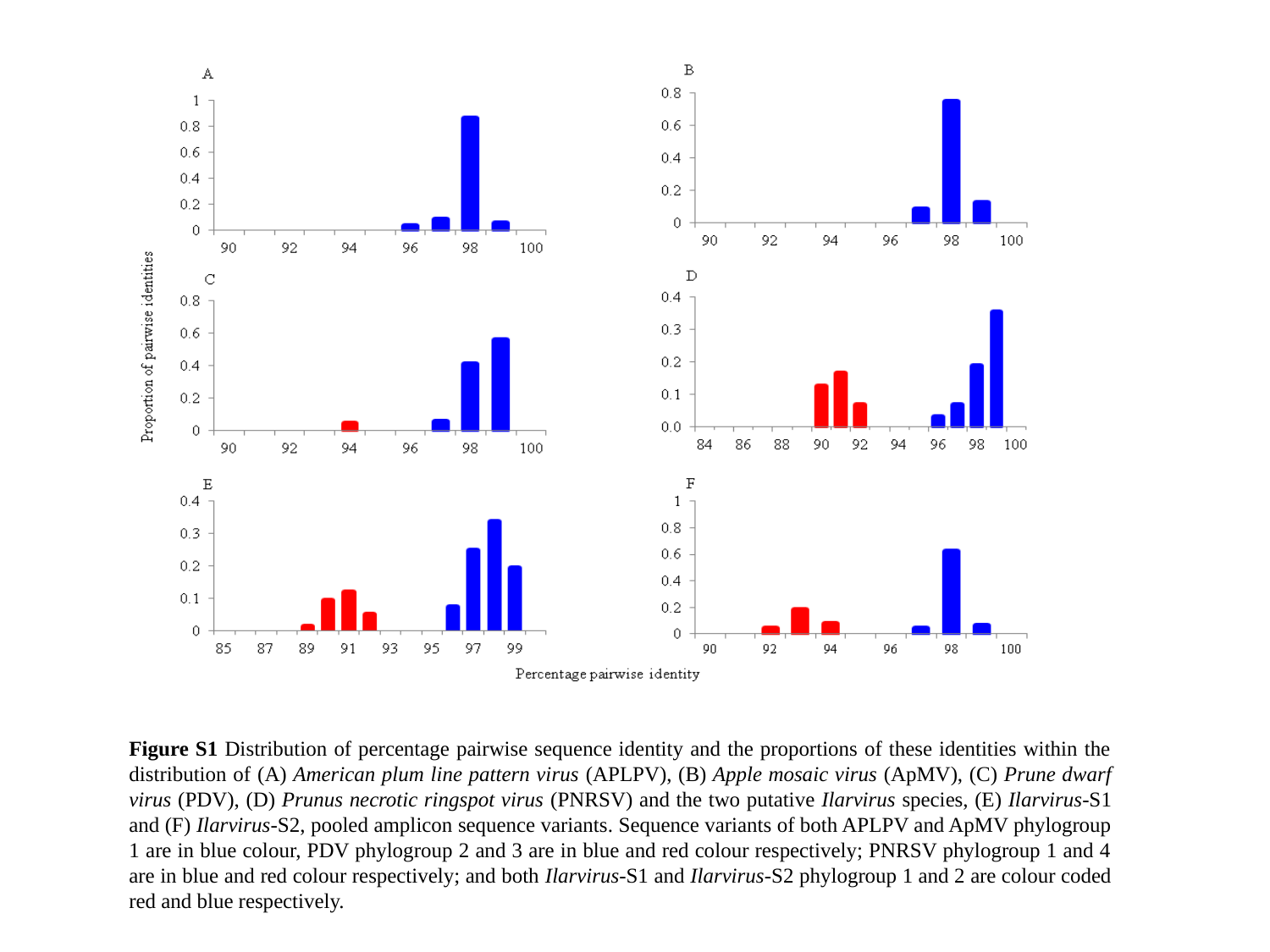

Figure S1 Distribution of percentage pairwise sequence identity and the proportions of these identities within the distribution of (A) American plum line pattern virus (APLPV), (B) Apple mosaic virus (ApMV), (C) Prune dwarf virus (PDV), (D) Prunus necrotic ringspot virus (PNRSV) and the two putative Ilarvirus species, (E) Ilarvirus-S1 and (F) Ilarvirus-S2, pooled amplicon sequence variants. Sequence variants of both APLPV and ApMV phylogroup 1 are in blue colour, PDV phylogroup 2 and 3 are in blue and red colour respectively; PNRSV phylogroup 1 and 4 are in blue and red colour respectively; and both Ilarvirus-S1 and Ilarvirus-S2 phylogroup 1 and 2 are colour coded red and blue respectively.
